# *Bifidobacterium pseudolongum* Strain PV8-2, Isolated from a Stool Sample of an Anemic Kenyan Infant

**DOI:** 10.1128/genomeA.01469-14

**Published:** 2015-01-22

**Authors:** Pamela Vazquez-Gutierrez, Christophe Lacroix, Christophe Chassard, Jochen Klumpp, Marc J. A. Stevens, Christoph Jans

**Affiliations:** a Laboratory of Food Biotechnology, Institute of Food, Nutrition and Health, ETH Zurich, Zurich, Switzerland; b Laboratory of Food Microbiology, Institute of Food, Nutrition and Health, ETH Zurich, Zurich, Switzerland

## Abstract

The complete genome sequence of Bifidobacterium pseudolongum PV8-2, isolated from feces of an anemic Kenyan infant, was determined using single-molecule real-time (SMRT) technology. The genome consists of a 2-Mbp chromosome and a 4-kb plasmid.

## GENOME ANNOUNCEMENT

Bifidobacteria are among the main anaerobic bacteria colonizing the gut of breastfed infants (1). The most predominant Bifidobacterium species in the infant gut are Bifidobacterium breve, Bifidobacterium longum, and Bifidobacterium bifidum, which represent about 80% of the total bifidobacterial count (2). Bifidobacterium pseudolongum belongs to the less predominant and less studied bifidobacteria in infants and accounts for approximately 2% of the bifidobacterial population colonizing the gut of infants (3). B. pseudolongum PV8-2 was isolated from the feces of an anemic Kenyan infant obtained during an iron intervention study (4), as described previously (5). This strain was selected after a thorough screening for potential probiotic bacteria of bifidobacterial isolates from our laboratory and public culture collections (5). Genomic DNA was prepared using a lysozyme/mutanolysine-based cell lysis and subsequent purification using the Wizard genomic DNA purification kit (Promega, Madison, WI, USA) (6). The genome was sequenced using 3 single-molecule real-time (SMRT) cells on a PacBio RS II (Pacific Biosciences, Menlo Park, CA, USA) and was performed at the Functional Genomics Center Zurich (Zurich, Switzerland). A total of 135,255 reads, with an average length of 4,506 bp, were assembled into a single contig using the hierarchical genome-assembly process (7). The genome was automatically annotated using the NCBI Prokaryotic Genomes Automatic Annotation Pipeline. Annotations were manually curated using respective annotations determined by the Rapid Annotation using Subsystems technology (RAST) platform (8). The genome of B. pseudolongum PV8-2 consists of a 2,032,698-bp circular molecule and comprises 53 tRNA genes and 4 rRNA operons encoding 12 rRNA genes. The G+C content of the genome is 63.3%, and a total of 1,582 protein coding sequences (CDS) were predicted. Additionally, a 4,251-bp plasmid with a G+C content of 53.6% was identified and harbored 5 CDS.

B. pseudolongum PV8-2 is the first infant B. pseudolongum species genome that has been completely sequenced and assembled. The genome will contribute to the understanding of genus evolution and adaptation to the gastrointestinal tract. It will provide further insights into the metabolism of bifidobacteria.

### Nucleotide sequence accession numbers.

The genome sequence of B. pseudolongum PV8-2 was deposited in GenBank under the accession number CP007457. The plasmid sequence of B. pseudolongum PV8-2 was deposited in GenBank under the accession number CP007458.
